# Supplementary material for: Incorporating evaporative water loss into bioenergetic models of hibernation to test for relative influence of host and pathogen traits on white-nose syndrome
Source: PLoS One. 2019 Oct 31;14(10):e0222311. doi: 10.1371/journal.pone.0222311 (PMC6822741; doi:10.1371/journal.pone.0222311)
Supplement: S1 File — Additional methodology regarding determining parameters for the hibernation energetics model and respirometry measurements. (DOCX) [file pone.0222311.s001.docx]

**S1 File**. **Supporting Information**. Additional methodology regarding determining parameters for the hibernation energetics model and respirometry measurements.

**Hibernation energetic model**

We modeled energy expended during a torpor-arousal bout (E_bout_) as a function of the energetic cost of torpor (E_tor_) and torpor bout duration (t_tor_), cost of euthermia (E_eu_) and euthermic duration per arousal bout (t_eu_), and the energy required to warm (E_warm_) and cool (E_cool_) to and from euthermia:

$E_{bout}= E_{eu} \cdot t_{eu} + E_{warm} +E_{cool} + E_{tor} \cdot t_{tor}$ (S1)

While bats are euthermic, the amount of energy expended is dependent on their resting metabolic rate; bats lose heat to the environment as a function of the differential between ambient temperature (T_a_) and the lower critical temperature (T_lc_), and the thermal conductance of euthermic tissue (C_eu_). Therefore, the energetic cost of euthermia (E_eu_) is essentially the basal metabolic rate (BMR), plus any additional cost due to temperatures outside the thermoneutral zone:

$E_{eu} = BMR + \left( T_{lc} - T_{a} \right) \cdot C_{eu}$ (S2)

We estimated BMR (ml O_2_ g^-1^ h^-1^) as a function of body mass (M_b_ in g) from a scaling equation derived for bats [1]:

${log}_{e}BMR = 1.0895 + 0.744{log}_{e}M_{b}$ (S3)

We estimated C_eu_ (ml O_2_ g^-1^ C^-1^ h^-1^) using methodology described by McNab [2] and estimated the mean time in euthermia during each arousal (t_eu_) from the published literature [3].

When bats arouse from torpor, they rewarm tissues from temperatures near or at T_a_. We therefore estimated the energetic cost of warming (E_warm_) as a function of the energy required to warm from torpid body temperature (T_tor_) to euthermic body temperature (T_eu_) given the specific heat of tissue (S; ml O_2_ g^-1^ C^-1^) and the amount of heat lost to the environment over the period of arousal as a defined by warming rate (WR; °C h^-1^ [4]):

$E_{warm}=S\cdot\left( T_{eu}-T_{torMin} \right)+ \frac{T_{eu}-T_{tor}}{WR}\left[ C_{eu}\cdot\left( T_{eu}-T_{a} \right) \right]$ (S4)

where T_tor_ is assumed to be at T_a_ unless hibernaculum temperature is below the lower defended torpid body temperature (T_tor-min_); below this temperature, T_tor_ remains at T_tor-min_^.^

We estimated the cost of cooling (E_cool_) as the decrease in metabolic rate over the time it takes to cool from euthermic to torpid body temperature, as a function of the cooling rate (CR;[5]):

$E_{cool}=\frac{\ln\left( T_{eu}-T_{torMin} \right)}{CR}\left[ {TMR}_{min}+\left( RMR \cdot{Q_{10}}^{\frac{T_{tor}-T_{eu}}{10}} \right) \right]$ (S5)

where TMR_min_is the minimum torpid metabolic rate (ml O_2_ g^-1^ h^-1^) and Q_10_ is the change in metabolism with a 10°C change in temperature [6]. We used Newton’s Law of Cooling to model cooling rate as a function of body mass, euthermic thermal conductance, and T_a_ [5]:

$CR=\frac{C_{eu} \cdot M_{b}^{0.67}\cdot\ln\left( T_{eu}-T_{a} \right)}{S\cdot M_{b}}$ (S6)

We modeled the energetic costs of torpor (E_tor_) as a function of T_a_ and torpid metabolic rate. When hibernaculum temperature is above minimum defended torpor temperature, energy expenditure can be calculated based on the Q_10_ effect [6]. Once hibernaculum temperature falls below the minimum defended temperature, E_tor_depends on torpid metabolic rate and torpid thermal conductance (C_t_):

$E_{tor} = {TMR}_{min} \cdot Q_{10}^{\left( T_{a}- T_{torMin} \right)/{10}}, if T_{a} > T_{torMin}$ (S7)

$E_{tor} = {TMR}_{min} + \left( T_{torMin}- T_{a} \right) \cdot C_{t}, if T_{a}\leq T_{torMin}$ (S8)

**Respirometry measurements**

All respirometry methodology is described in detail in Fuller et al. [7]. Using open flow respirometry [8], we measured torpor metabolic rate (TMR) and evaporative water loss (EWL) across multiple temperatures. Bats were placed in individual chambers in a dark temperature controlled cabinet at 10°C for approximately 12 hours until they returned to torpor following capture. EWL was measured for all bats at 10 °C, controlling relative humidity with a dew point generator (DG-4; Sable Systems) at 0%; excurrent water vapor pressure was measured with a humidity meter (RH-300; Sable Systems) to calculate EWL. We then reduced the temperature of the chamber to 8 °C, 5 °C, and 2 °C for three hours each to record TMR at a variety of temperatures. Respirometry flow rate was controlled with an air flow manifold (FB-8; Sable Systems) and respirometry data was collected from each bat using a multiplexer (RM-8; Sable Systems), continuously rotating among bats for the duration of the respirometry measurements. TMR was calculated based on oxygen consumption (FC-10; Sable Systems) and carbon dioxide production (CA-10; Sable Systems) as per equations in Lighton [8]. We found no difference in TMR between fall and winter seasons; we therefore used the mean of the minimum TMR per individual across seasons to use in our model (Table 1).

**Estimation of surface area and calculation of rEWL**

We calculated surface area by first using a common scaling equation to estimate surface area from mean body mass [9]:

${SA}_{body}=10 \cdot{M_{b}}^{\frac{2}{3}}$ (S1)

We then considered the extra surface area associated with bat wings and tails (SA_wing_) by using image processing software (ImageJ; <https://imagej.nih.gov/ij/>) to calculate a percent increase to surface area due to the area of bat wing and tails that are exposed to the environment. We obtained photos of *M. lucifugus* roosting on cave walls where the forearm and tail were visible, and then set the scale using the mean forearm length measured from our field data. We measured the approximate area of the folded wing and tail, and calculated a percent increase to SA_body_ values estimated by Equation S1.

We used two different rates of area-specific cutaneous evaporative water loss (rEWL): one for the body surface area (rEWL_body_), and one for the wing and tail (rEWL_wing_). We calculated rEWL_body_ from measurements of EWL from small mammals (e.g. mice) found in the literature [10], assuming that fur on the body of bats and from small mammals had the same properties that would influence EWL. Studies have also shown that hibernating mammals lose most body water through the cutaneous membranes, given respiration rate is so low during torpor. Therefore we assumed that 99.7% of EWL values measured during torpor represented cutaneous EWL [11]. We rearranged Equation 5 to solve for rEWL_body_ given the hourly EWL, air temperature, and relative humidity reported in each publication; we calculated SA_body_ using Equation S1 and the body mass reported in each publication. We took the mean rEWL_body_ across species and temperatures to use for our bat rEWL_body_.

We then calculated rEWL_wing_ by using our measured EWL from the field. We first calculated the proportion of cutaneous EWL (99.7% of total EWL) from the body using Equation 5, given rEWL_body_, surface area (calculated as described above), and the air temperature and water vapor pressure measured for each metabolic chamber. We subtracted this value from the EWL measured, and rearranged Equation 5 as described above to solve for rEWL_wing_. We took the mean rEWL_wing_ to use for the rest of our calculations.

**Estimation of increase to rEWL_wing_ and TMR due to fungal growth**

We calculated the increase in rEWL_wing_ due to fungal growth by using the data presented by McGuire et al. [12]. We used Equation 5 to solve for rEWL_wing_ for the reported measurements of EWL (see Figure 3a in McGurie et al. [12]) across % surface area of the wing covered in *P. destructans* lesions, and took the slope of that relationship to represent the linear increase in rEWL_wing_ due to fungal growth.

To calculate the linear increase to torpid metabolic rate due to fungal growth, we used the metabolic rate data from wet conditions presented in Table 1 in McGuire et al. [12]. We predicted fungal growth at the end of the experiment (113 days) using equations from Hayman et al. [13] and estimated the percent of bat wing surface area (calculated as SA_wing_ above) covered in *P. destructans*. We then determined the relationship between TMR and fungal growth: we treated the control measurement as 0 fungal growth and used the predicted fungal growth at day 113, and took the slope of this relationship to represent the linear increase of TMR to the proportion of the bat wing covered in fungal growth.

**Calculation of threshold of water loss**

Previous models have suggested that bats remain in torpor until a threshold of total body water is reached [11,14,15]. The threshold of total body water previously used was estimated to be 4.3% of a bat’s lean mass; however, this value was estimated from a single study and may not represent inter- and intraspecific variation. Therefore, we collected torpor bout duration and EWL data on multiple species and back-calculated threshold values (Supplementary Table S1). More specifically, we rearranged Equation 9 to solve for the percentage of lean mass that determines the total body water threshold given reported torpor bout duration and EWL, so that:

$\text{Threshold \%}\text{ = }\frac{\text{TBD * EWL}}{\text{Lean mass *}\text{ }\text{1000}}$ (S2)

where TBD is the torpor bout duration (h), EWL is the hourly rate of evaporative water loss (mg H_2_O h^-1^), and lean mass is in grams. If torpor bout duration was reported but EWL was not, we calculated EWL given equations in the main text and bat morphometric data and hibernaculum conditions reported in each publication. The percent of lean mass threshold ranged from 1.6% to 4.7%; therefore, we determined that, on average, bats can lose 2.7% of their lean mass in mg of water before arousing.

**
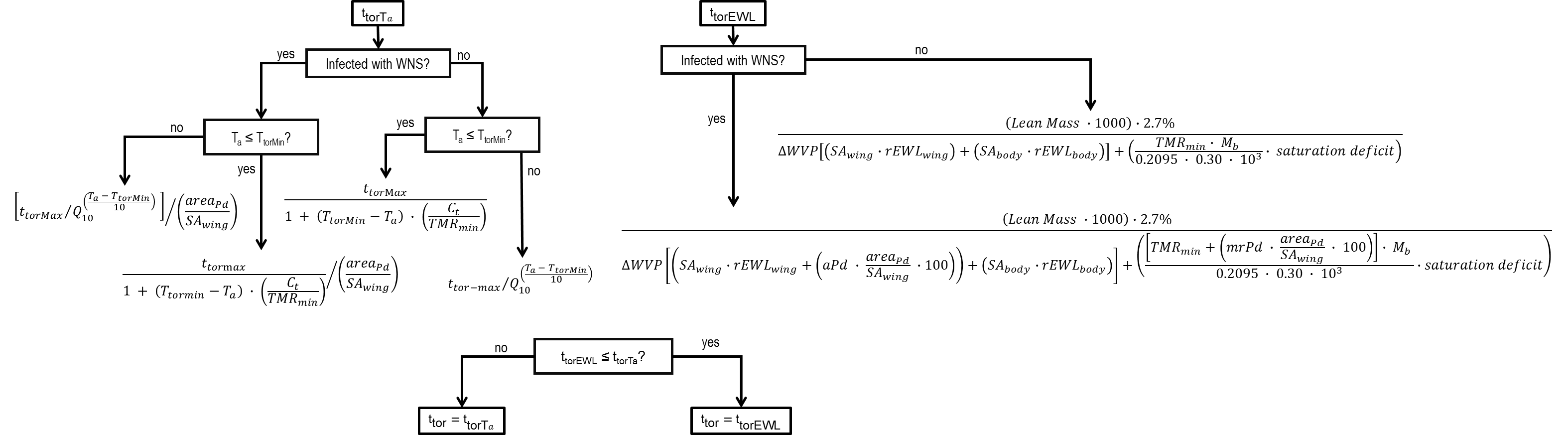
**

**Fig A. Flow diagram of the calculation of torpor bout duration (t_tor_) in the bioenergetic model.** If body water threshold is not met by evaporative water loss, t_tor_ is determined by metabolic response to hibernaculum temperature (t_torTa_); otherwise t_tor_ is determined by the time to reach body water threshold given T_a_ and relative humidity (t_torEWL_). All parameters are described in Table 1


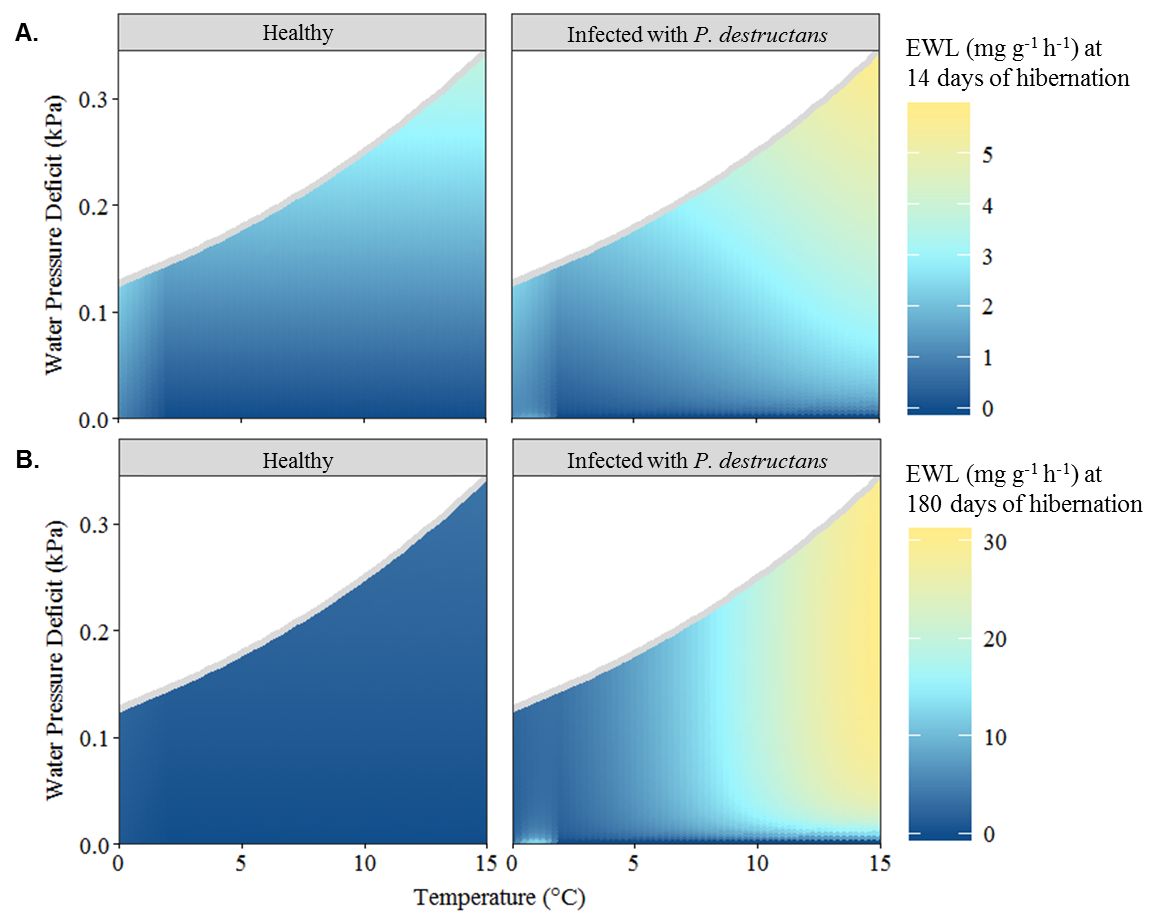


**Fig B. Predictions from the EWL model.** Predicted rate of total evaporative water loss (EWL; mg H_2_O g^-1^ h^-1^) from *Myotis lucifugus* for both healthy bats and bats infected with *P. destructans* at the (**A**) start (14 days) and (**B**) end (180 days) of hibernation over a range of temperature and water pressure deficit conditions. White area bounded by grey line represents impossible parameters space for each temperature; i.e. the saturation of air at each temperature when it cannot hold more water.

**
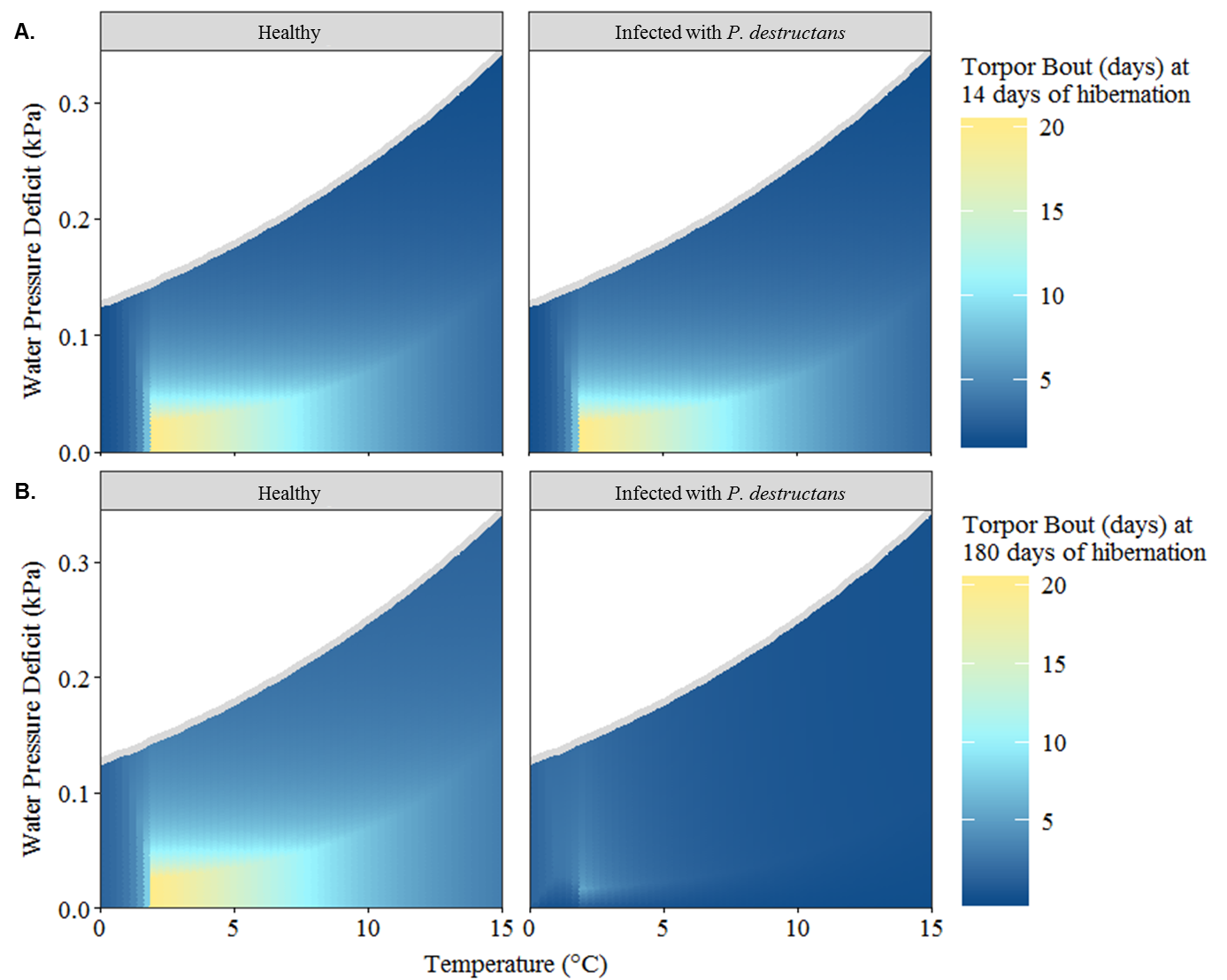
**

**Fig C. Predictions from the torpor bout duration model.** Predicted torpor bout duration (days) from *Myotis lucifugus* for both healthy bats and bats infected with *P. destructans* at the (**A**) start (14 days) and (**B**) end (180 days) of hibernation over a range of temperature and water pressure deficit conditions. White area bounded by grey line represents impossible parameters space for each temperature; i.e. the saturation of air at each temperature when it cannot hold more water.

**Table A.** Measurements of torpor bout duration (TBD) and evaporative water loss (EWL) from the literature used to calculate the total body water threshold percentage to determine arousal events.

| Species | Mass (mg) | Lean Mass (mg) | Temperature (C) | EWL (mg h^-1^) | TBD (h) | Reference |
| --- | --- | --- | --- | --- | --- | --- |
| *Spermophilus saturatus* | 250000 | 200000 | -2 | 22.24 | 204.48 | [15] |
| *Spermophilus saturatus* | 250000 | 200000 | 2 | 20.53 | 255.6 | [15] |
| *Spermophilus saturatus* | 250000 | 200000 | 4 | 21.80 | 199.44 | [15] |
| *Spermophilus saturatus* | 250000 | 200000 | 8 | 28.70 | 111.12 | [15] |
| *Pipistrellus kuhlii* | 7280 | 5824 | 5 | 4.17 | 48 | [16] |
| *Pipistrellus kuhlii* | 7280 | 5824 | 5 | 2.08 | 132 | [16] |
| *Myotis lucifugus* | 8880 | 4700 | 7 | 0.15 | 211.90 | [17] |
| *Myotis lucifugus* | 8500 | 4500 | 7 | 0.23 | 196.14 | [17] |
